# Supplementary material for: Verproside, the Most Active Ingredient in YPL-001 Isolated from Pseudolysimachion rotundum var. subintegrum, Decreases Inflammatory Response by Inhibiting PKCδ Activation in Human Lung Epithelial Cells
Source: Int J Mol Sci. 2023 Apr 13;24(8):7229. doi: 10.3390/ijms24087229 (PMC10138391; doi:10.3390/ijms24087229)
Supplement: Supplementary file 1 [file ijms-24-07229-s001.zip › ijms-2171157-supplementary.pdf]

Supplementary Information

Verproside, the most active ingredient in the YPL-001 isolated from *Pseudolysimachion rotundum* var. *subintegrum*, decreases inflammatory response by inhibiting PKCδ activation in human lung epithelial cells

---

• Supplementary Figures S1 and S2

Figure S1-1 Preparative HPLC fractionation of *P. rotundum* var. *subintegrum* extract 2

Figure S1-2. UPLC-PDA of fractions of *P. rotundum* var. *subintegrum* extract 3

Figure S1-3. ^{1}H and ^{13}C NMR spectrum of Piscroside C (**1**) 4

Figure S1-4. ^{1}H and ^{13}C NMR spectrum of Verproside (**2**) 5

Figure S1-5. ^{1}H and ^{13}C NMR spectrum of Isovanillyl catalpol (**3**) 6

Figure S1-6. ^{1}H and ^{13}C NMR spectrum of Picroside II (**4**) 7

Figure S1-7. ^{1}H and ^{13}C NMR spectrum of 6-*O*-Veratroyl catalpol (**5**) 8

Figure S1-8. ^{1}H and ^{13}C NMR spectrum of Catalposide (**6**) 9

Figure S2-1. UPLC-QTof-MS chromatogram of *P. rotundum* var. *subintegrum* extract 11

Figure S2-2. UV, MS/MS, MS and HREIMS data of Piscroside C (**1**) 11

Figure S2-3. UV, MS/MS, MS and HREIMS data of Verproside (**2**) 12

Figure S2-4. UV, MS/MS, MS and HREIMS data of Isovanillyl catalpol (**3**) 12

Figure S2-5. UV, MS/MS, MS, and HREIMS data of Picroside II (**4**) 13

Figure S2-6. UV, MS/MS, MS and HREIMS data of 6-*O*-Veratroyl catalpol (**5**) 13

Figure S2-7. UV, MS/MS, MS and HREIMS data of Catalposide (**6**) 14

Figure S3. Original western blots in Figure 5 15

Figure S4. Original western blots in Figure 6 15

Figure S5. Molecular docking simulation of PKCδ and verproside 17

• Supplementary Table 1

- Table S1. Molecular docking study of PKCδ with six iridoids (**1-6**). 17

Experimental Section

1D (^1H, and ^{13}C) NMR spectra were obtained on JEOL ECZ500R (^1H NMR at 500 MHz, ^{13}C NMR at 125 MHz, Tokyo, JP), Varian UNITY 400 NMR(^1H NMR at 400 MHz, ^{13}C NMR at 100 MHz, PaloAlto, USA) and Bruker AVANCE III HD 700 (^1H NMR at 700 MHz, ^{13}C NMR at 175 MHz) using acetnone-d_6 (Cambridge Isotope Laboratories, Andover, MA) as an NMR solvent and tetramethylsilane (TMS) as an internal standard. HRESIMS were measured on an ultraperformance liquid chromatography quadrupole time-of-flight mass spectrometer (UPLC-QTof-MS, Waters, Milford, MA, USA) in the negative-ion mode. Preparative HPLC was performed with a K-Prep LAB-300G instrument (YMC, Kyoto, Japan). Semiprepartive HPLC separation (Gilson, Middleton, WI, USA) is comprised of a standard binary pump (321 HPLC pump), UV/Vis detector (172 DAD), evaporative light-scattering detector (ELSD, Varian 380-LC), and injection modules (GX271 liquid handler).

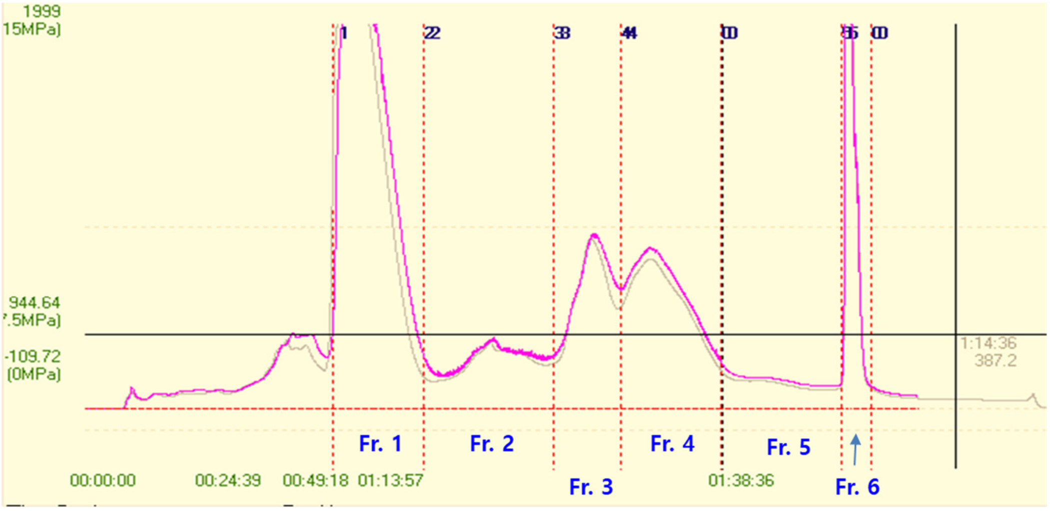

Condition

YPL_001	
Instrument	YMC_LAB
Guard Column	YMC-ODS-AQ-HG-10 um, 298 g<br>YMC-ODS-AQ-HG-10 um, 220 g
Solvents	MeOH/D.W
Flow Rate	150 ml/min
UV	254 nm
Loading sample	4 g/9 ml

Method - Gradient

Time(min)	%A	%B
(Initial)	73	27
10.00	73	27
60.00	65	35
61.00	0	100
76.00	0	100
77.00	73	27
97.00	73	27

Figure S1-1. Preparative HPLC fractionation of *P. rotundum* var. *subintegrum* extract (YPL-001).

UPLC method

① UPLC

Waters Acquity UPLC system

② Column

ACQUITY UPLC ® BEH C_{18} 1.7㎛ 2.1x100mm

③ Absorbance

254nm

④ Concentration : 1mg/ml

⑤ Injection volume : 3 µl

⑥ Gradients

Time(min)	Flow(mL/min)	%A(0.1% Formic acid/DW)	%B(0.1% Formic acid/ACN)
0.00	0.4	90	10
1.00	0.4	90	10
10.50	0.4	77	23
12.00	0.4	2	98
13.30	0.4	2	98
13.40	0.4	90	10
15.00	0.4	90	10

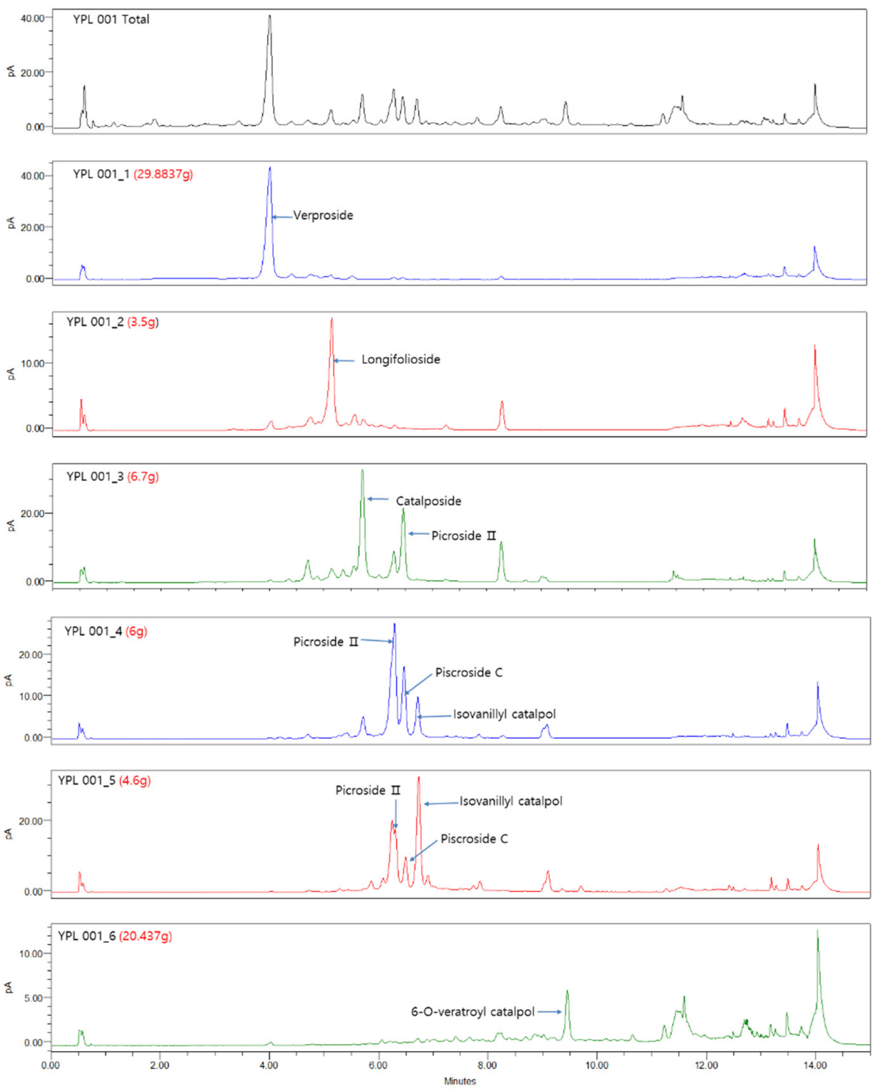

Figure S1-2. UPLC-PDA of fractions of *P. rotundum* var. *subintegrum* extract (YPL-001). YPL001_1: Fr.1 and Fr.2, YPL001_3: Fr.3, YPL001_4: Fr.4, YPL001_5: Fr.5, YPL001_6: Fr.6

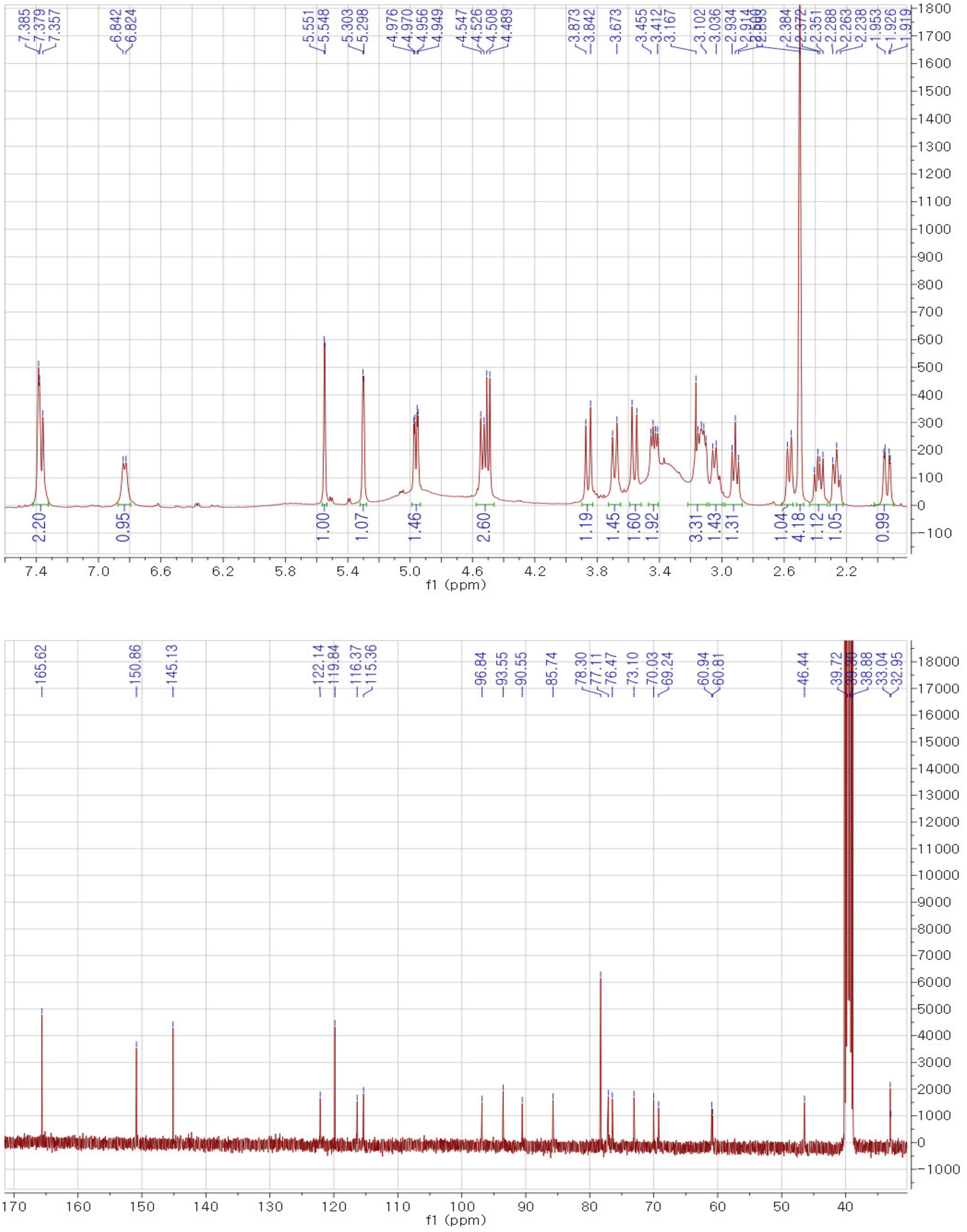

Figure S1-3. ^{1}H and ^{13}C NMR spectrum of Piscroside C (**1**).

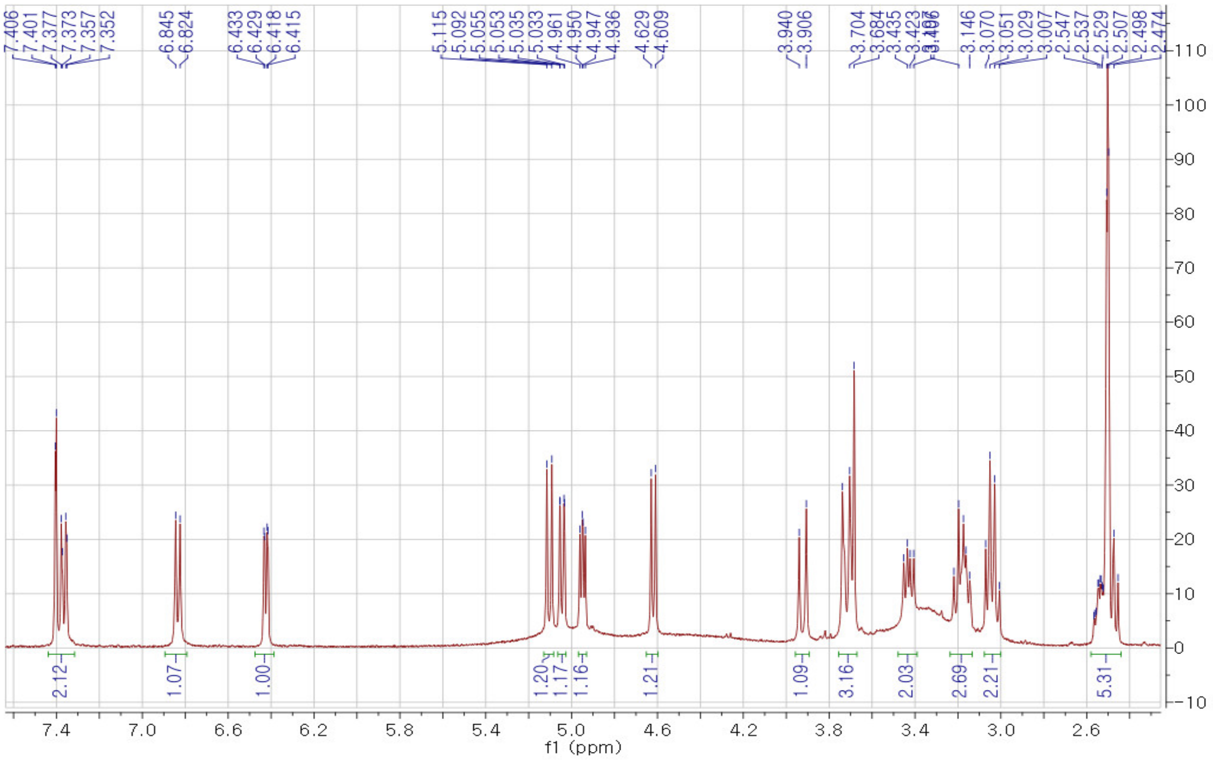

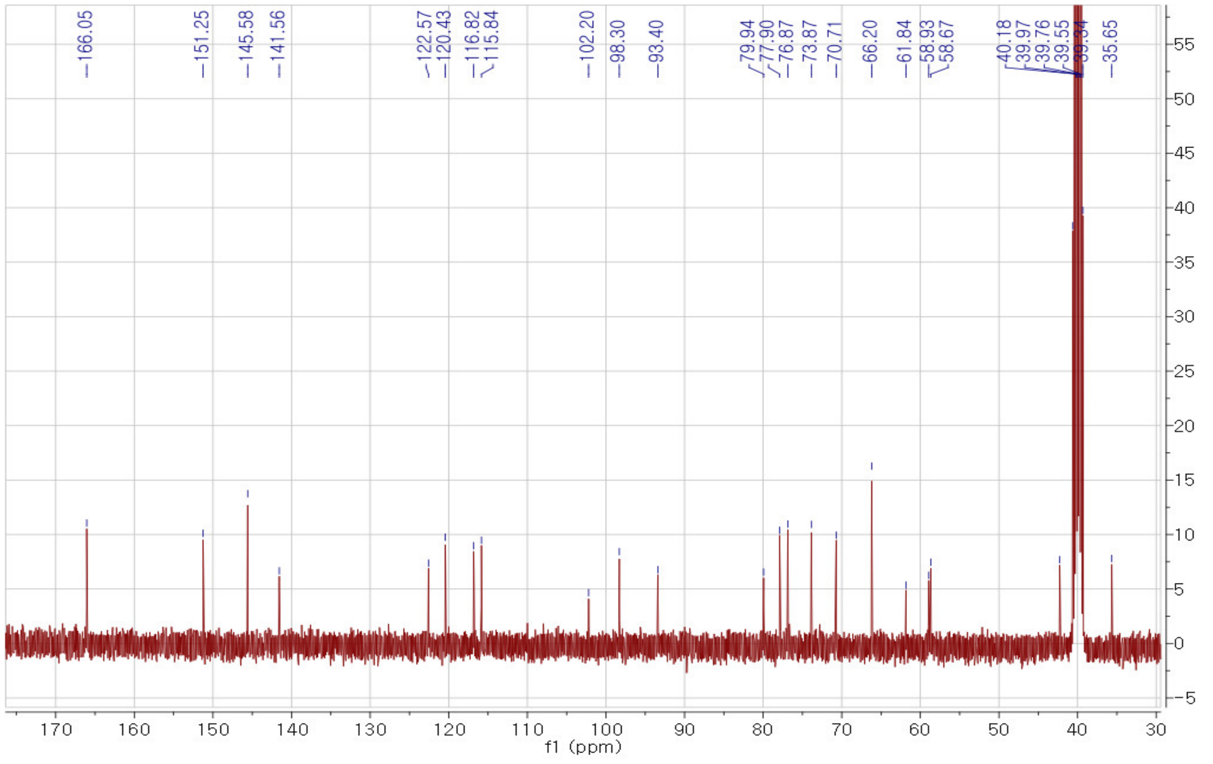

Figure S1-4. ^{1}H and ^{13}C NMR spectrum of Verproside (**2**).

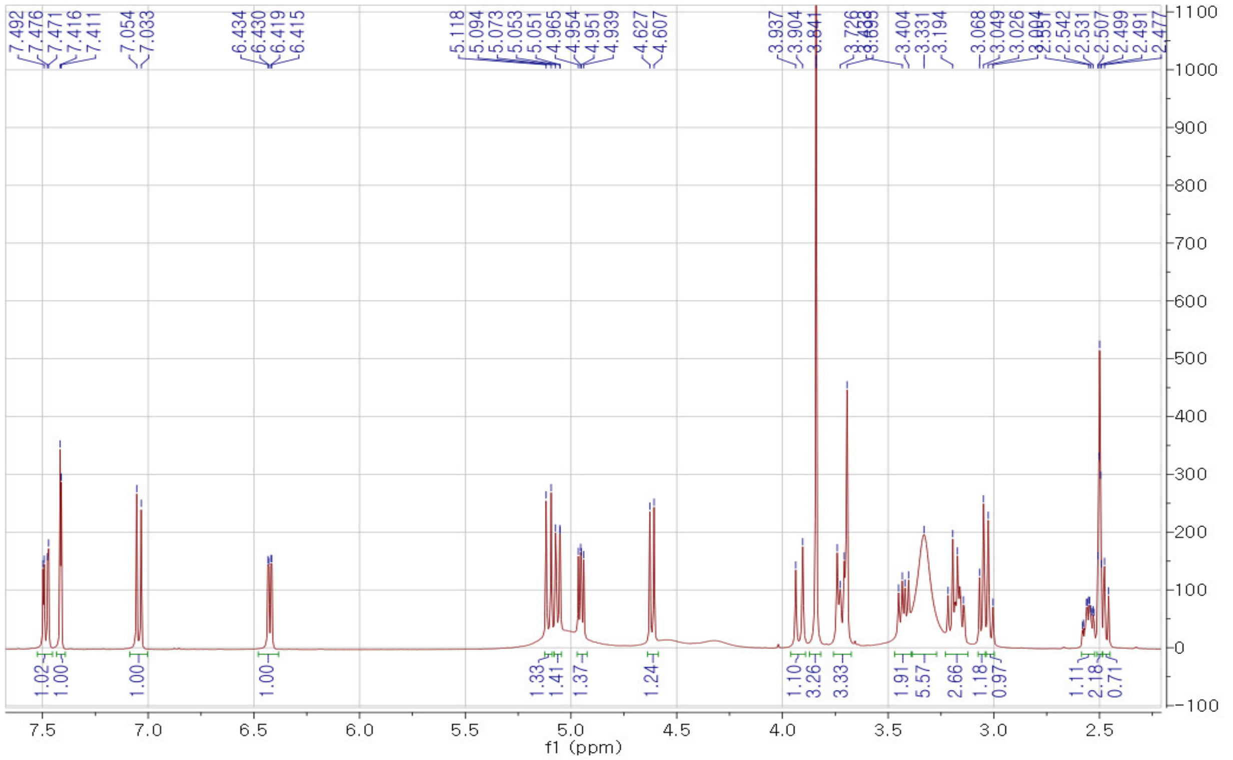

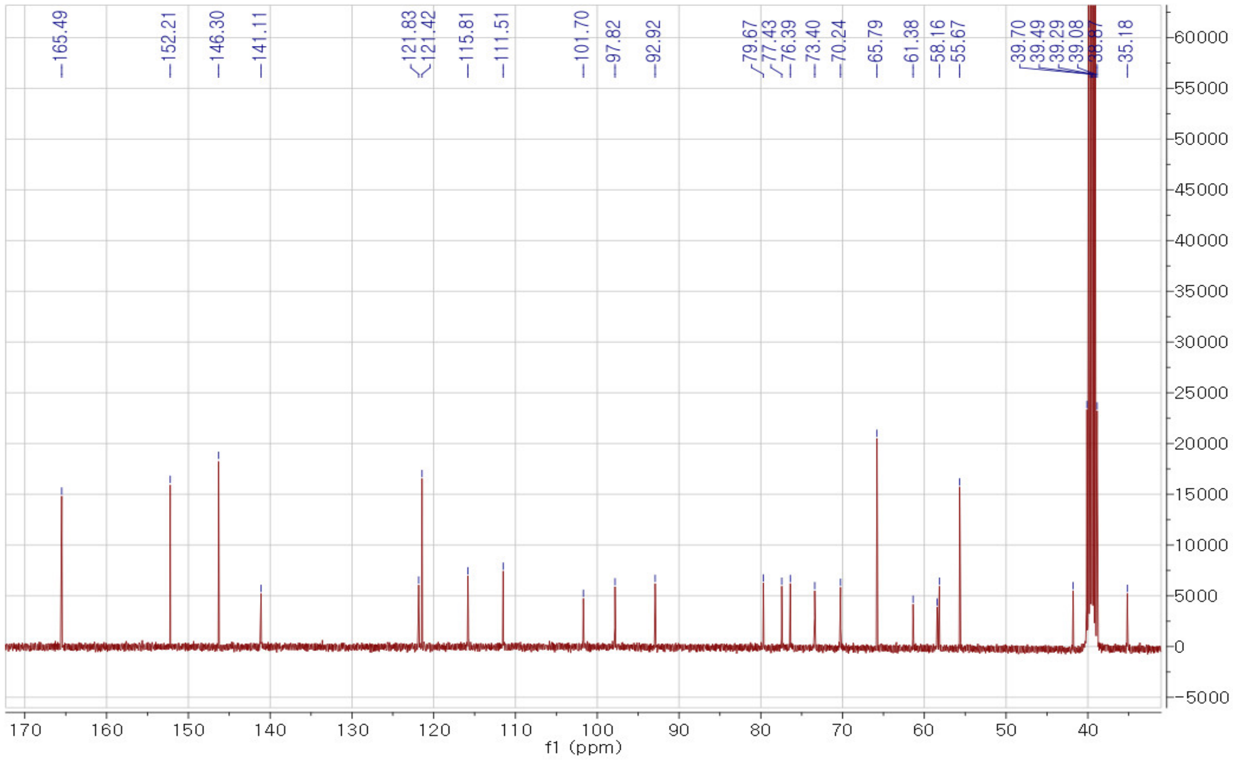

Figure S1-5. ^{1}H and ^{13}C NMR spectrum of Isovanillyl catalpol (**3**).

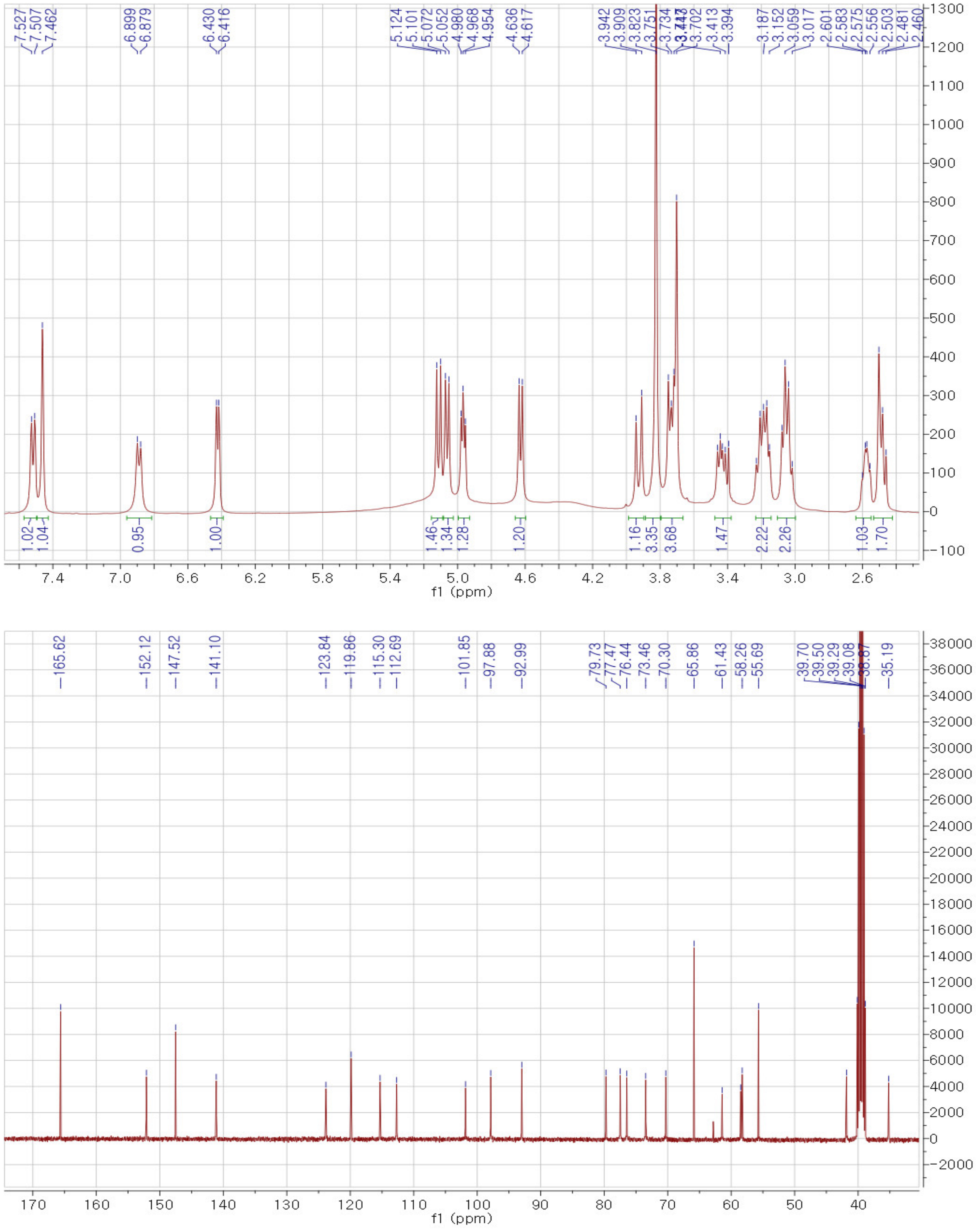

Figure S1-6. ^{1}H and ^{13}C NMR spectrum of Picroside II (**4**).

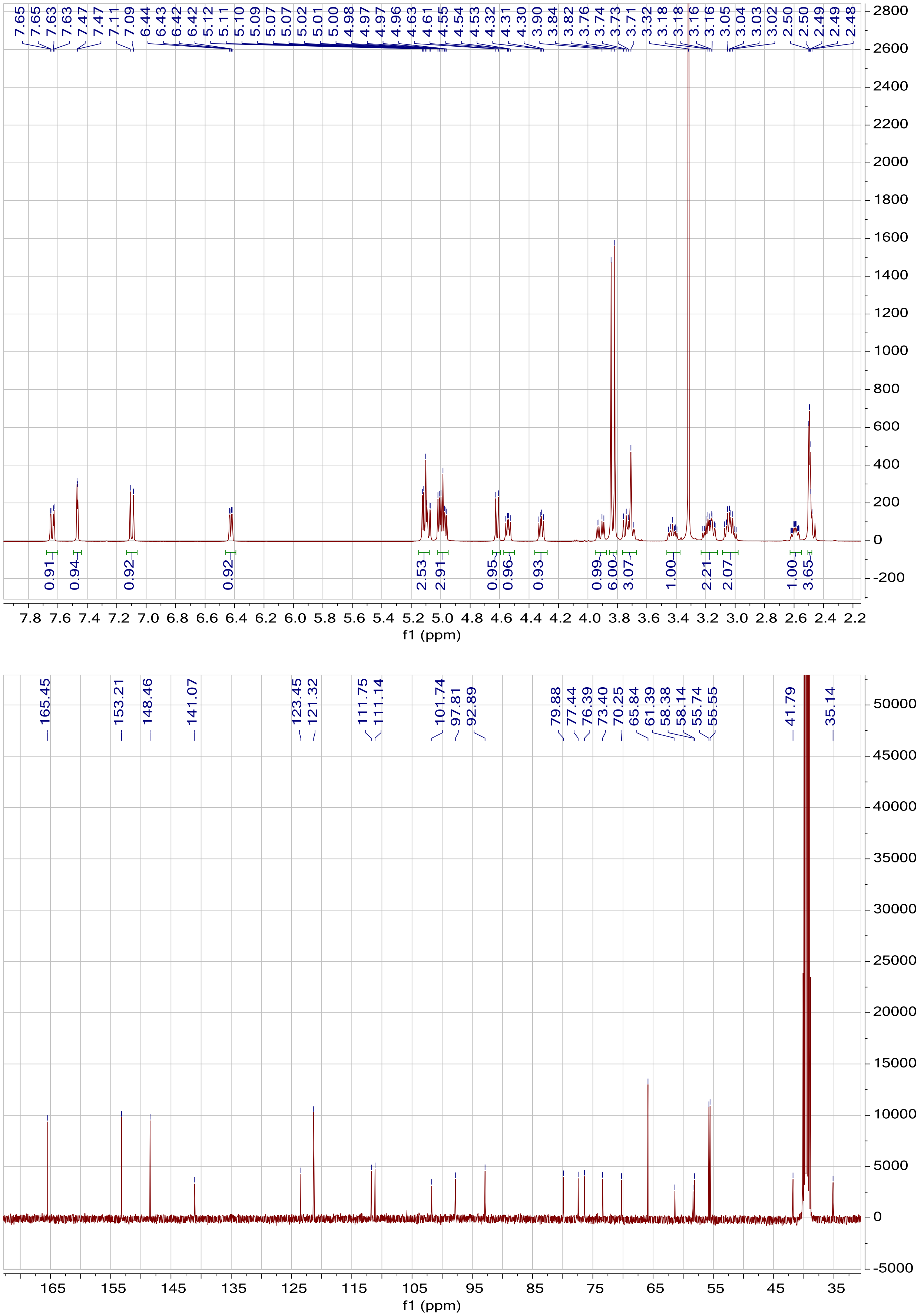

Figure S1-7. ^{1}H and ^{13}C NMR spectrum of 6-*O*-Veratroyl catalpol (**5**).

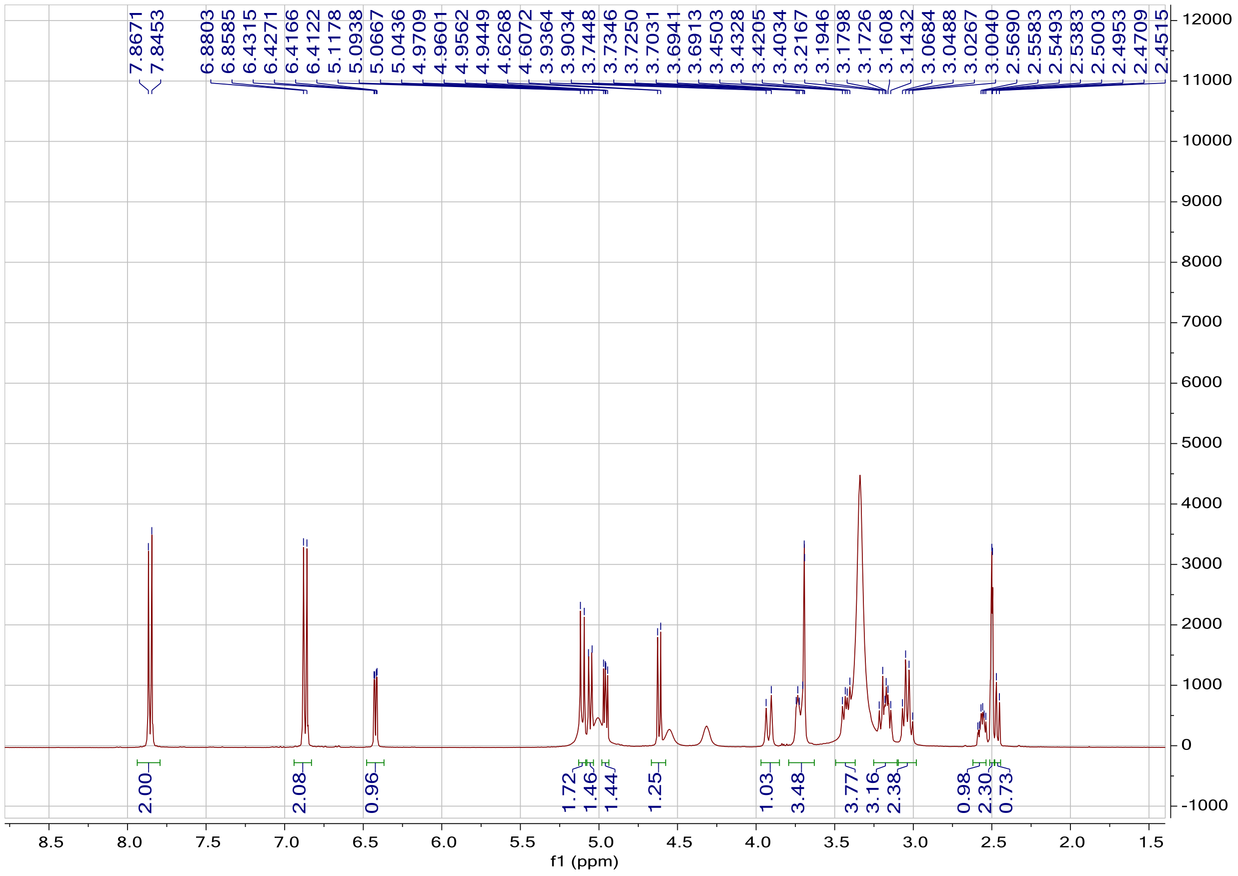

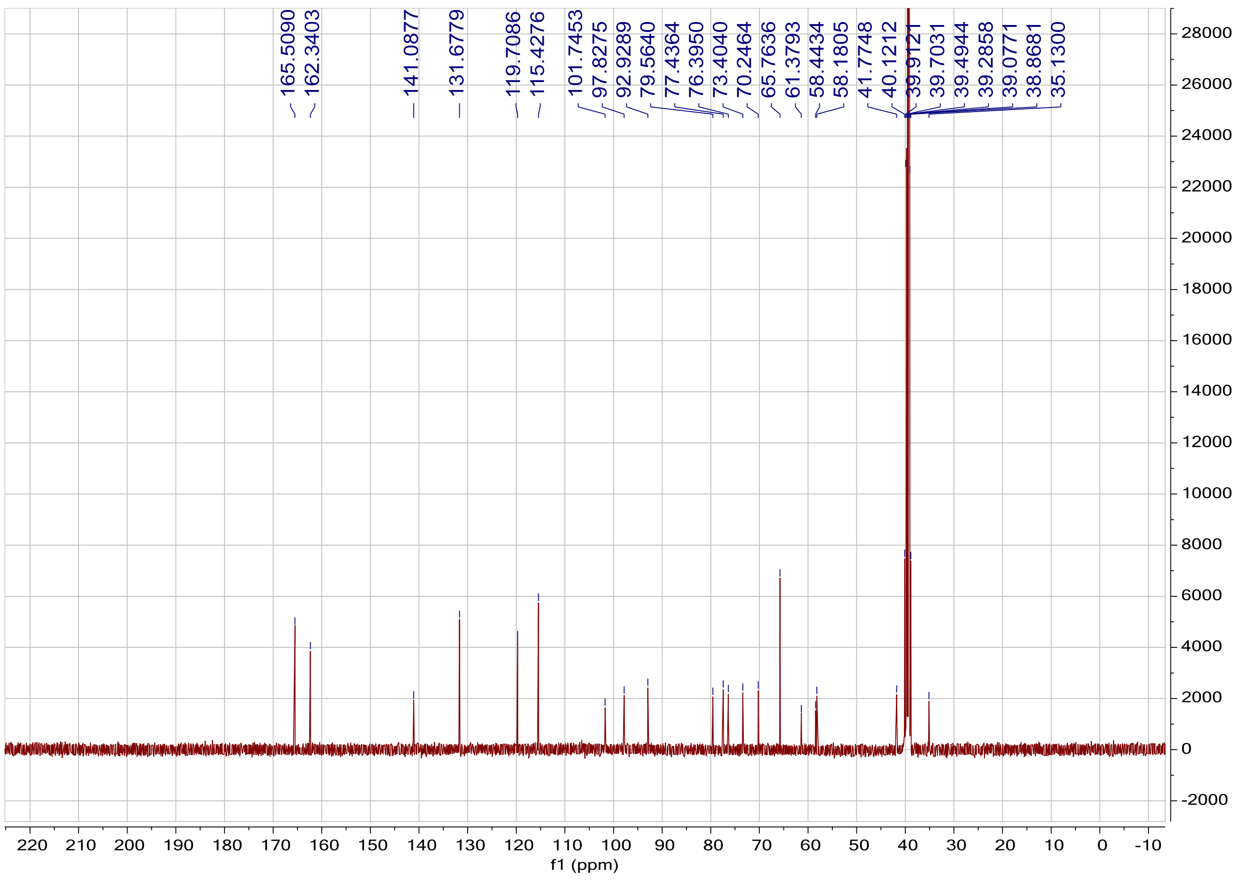

Figure S1-8. ^{1}H and ^{13}C NMR spectrum of Catalposide (**6**).

UPLC-QTof-MS analysis

P. rotundum var. *subintegrum* extract (YPL-001) profiling was performed using an ACQUITY UPLCTM system (Waters Corporation, Milford, MA, USA) equipped with a binary solvent delivery manager and a sample manager coupled to a Micromass Q-TOF Premier™ mass spectrometer (Waters Corporation) with electrospray ionization (ESI) interface with MassLynex V4.1 software. Chromatographic separation was performed using an ACQUITY BEH C18 chromatography column (2.1 × 100 mm, 1.7 μm). The column temperature was maintained at 35°C, and the mobile phases A and B were water with 0.1% formic acid and acetonitrile with 0.1% formic acid, respectively. The gradient elution program was as follows: 0.0-1.0 min, 10% B; 1.0-10.5 min, 10-23% B; 10.5-12.0 min, 23-98% B; wash for 1.4 min with 100% B; and a 1.6 min recycle time. The injection volume was 2.0 μL, and the flow rate was 0.4 mL/min. The mass spectrometer was operated in positive ion mode. N_2 was used as the desolvation gas. The desolvation temperature was 350°C, the flow rate was 500 L/h, and the source temperature was 110°C. The capillary and cone voltages were 2300 V and 50 V, respectively. The Q-TOF Premier™ was operated in v mode with a 9000 mass resolving power. The data were collected for each test sample from 100 to 1500 Da with 0.25-s scan time and 0.01-s interscan delay over the 15 min analysis time. Leucine-enkephalin was used as the reference compound (*m/z* 554.2661 in the negative mode).

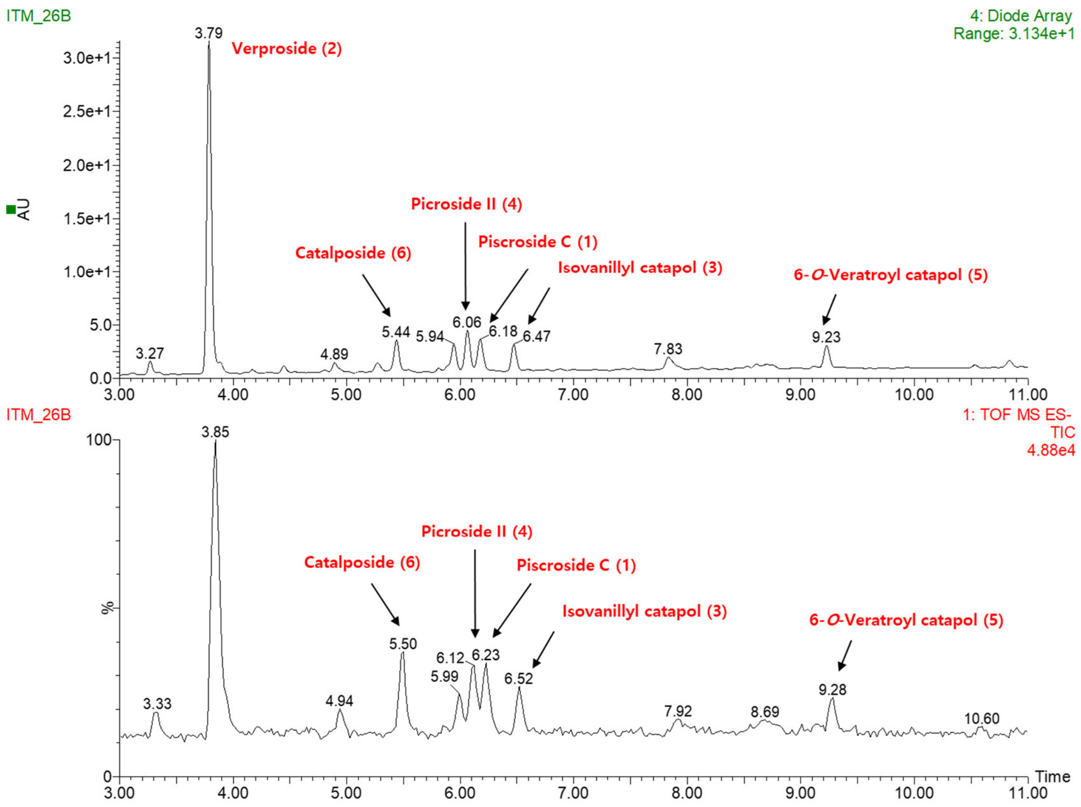

Figure S2-1. UPLC-QTof-MS chromatogram of *P. rotundum* var. *subintegrum* extract (YPL-001).

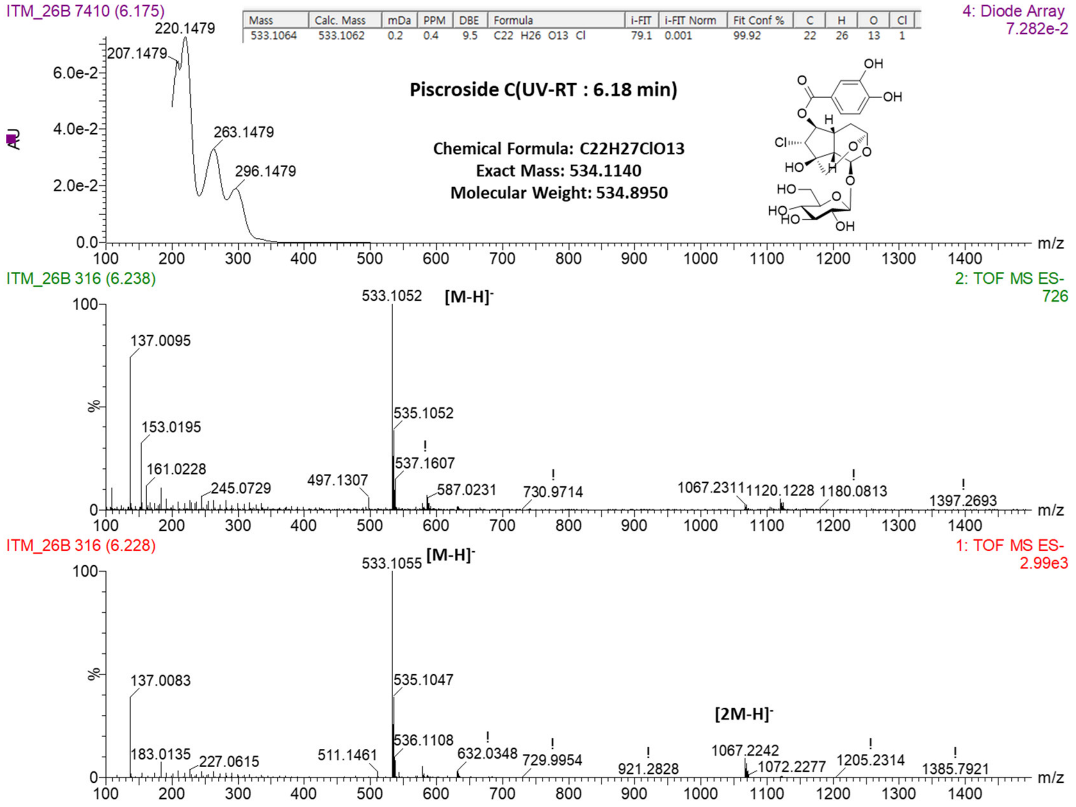

Figure S2-2. UV, MS/MS, MS and HREIMS data of Piscroside C (**1**).

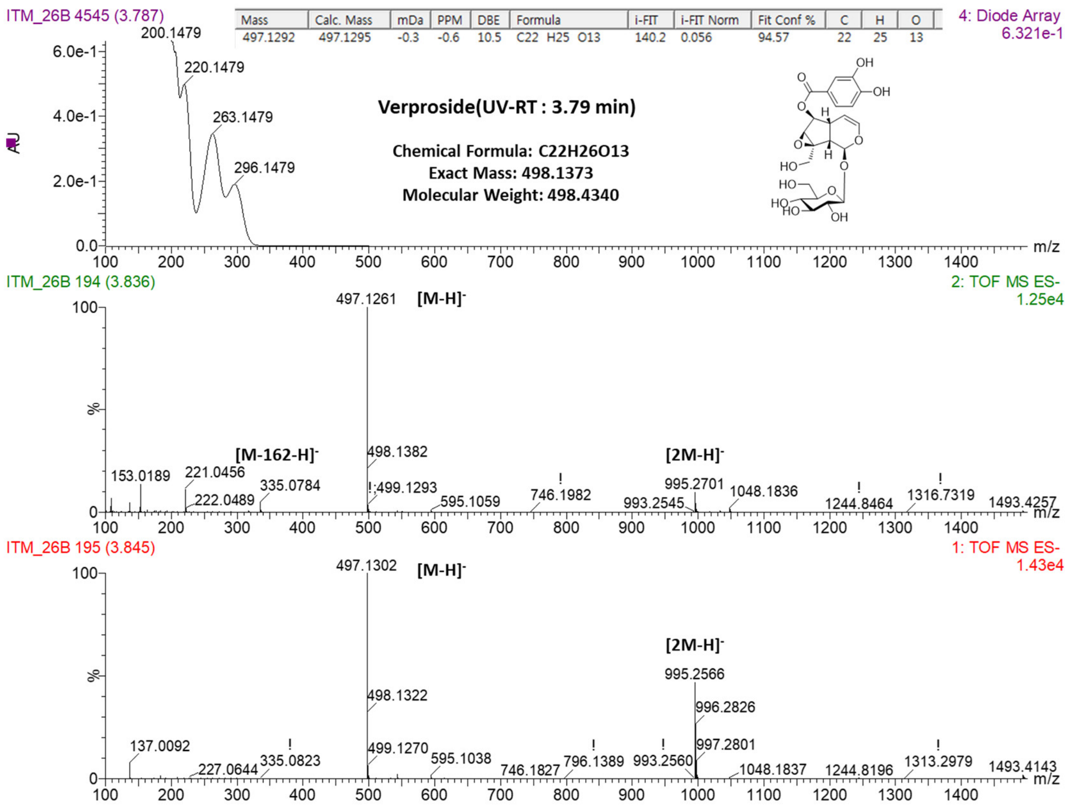

Figure S2-3. UV, MS/MS, MS and HREIMS data of Verproside (**2**).

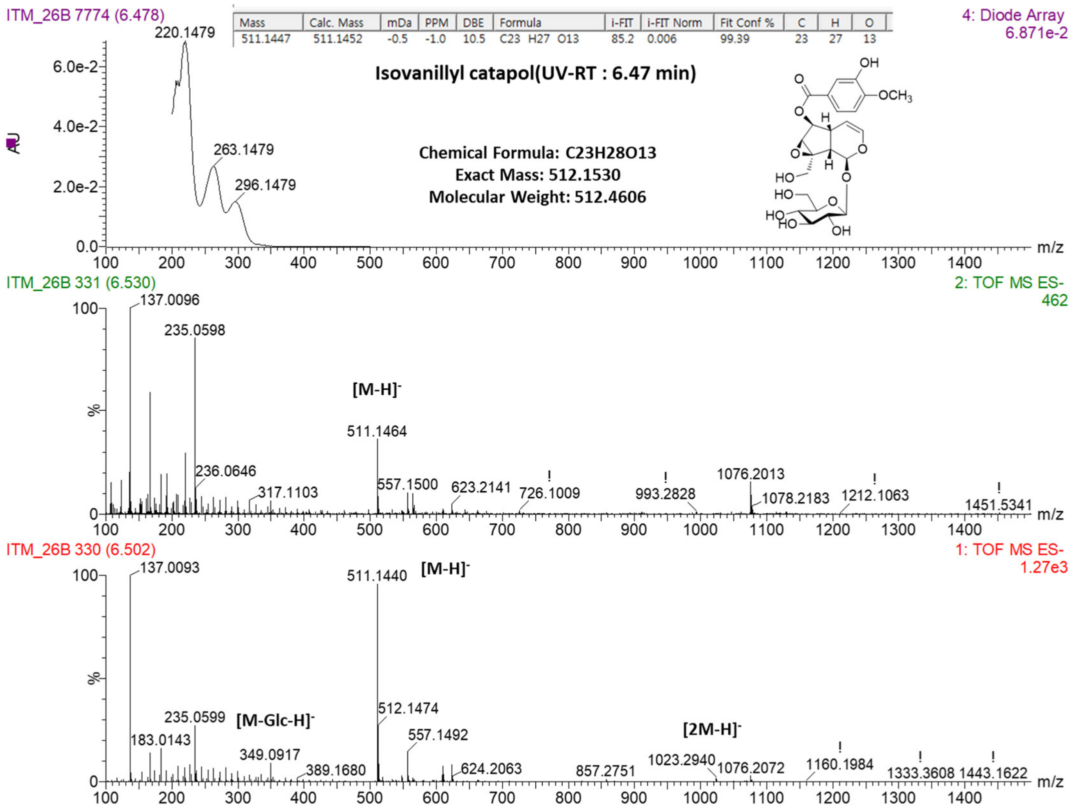

Figure S2-4. UV, MS/MS, MS and HREIMS data of Isovanillyl catalpol (**3**).

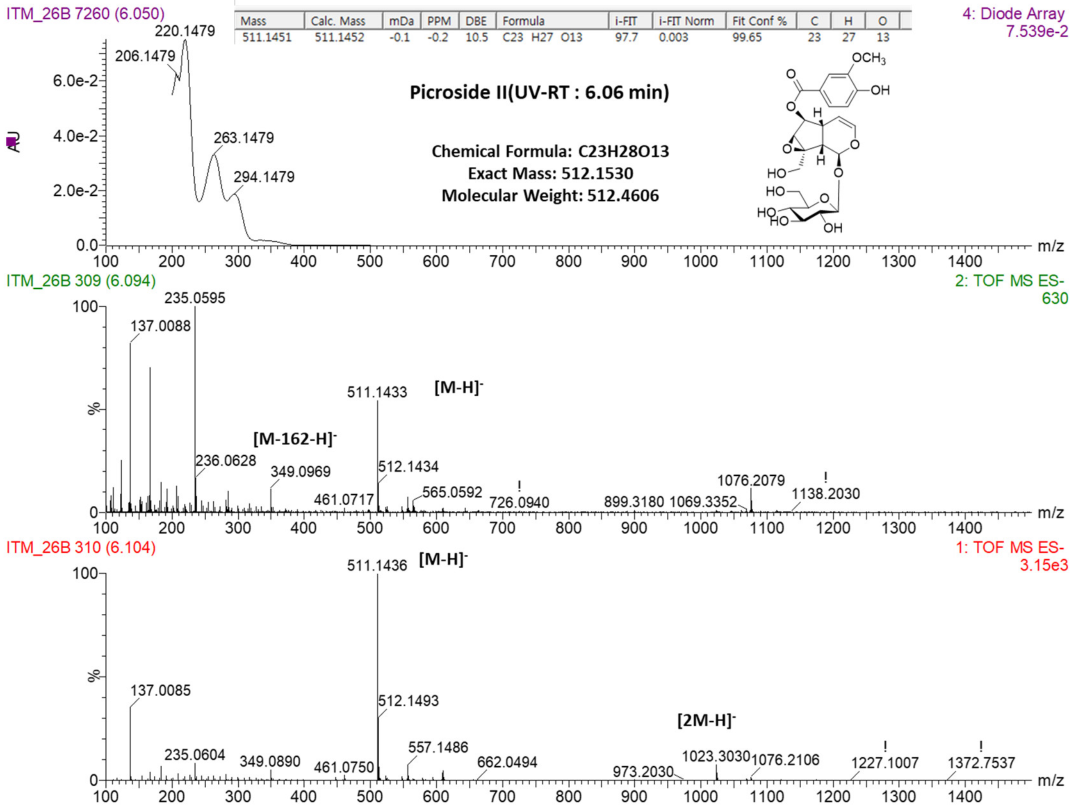

Figure S2-5. UV, MS/MS, MS and HREIMS data of Picroside II (**4**).

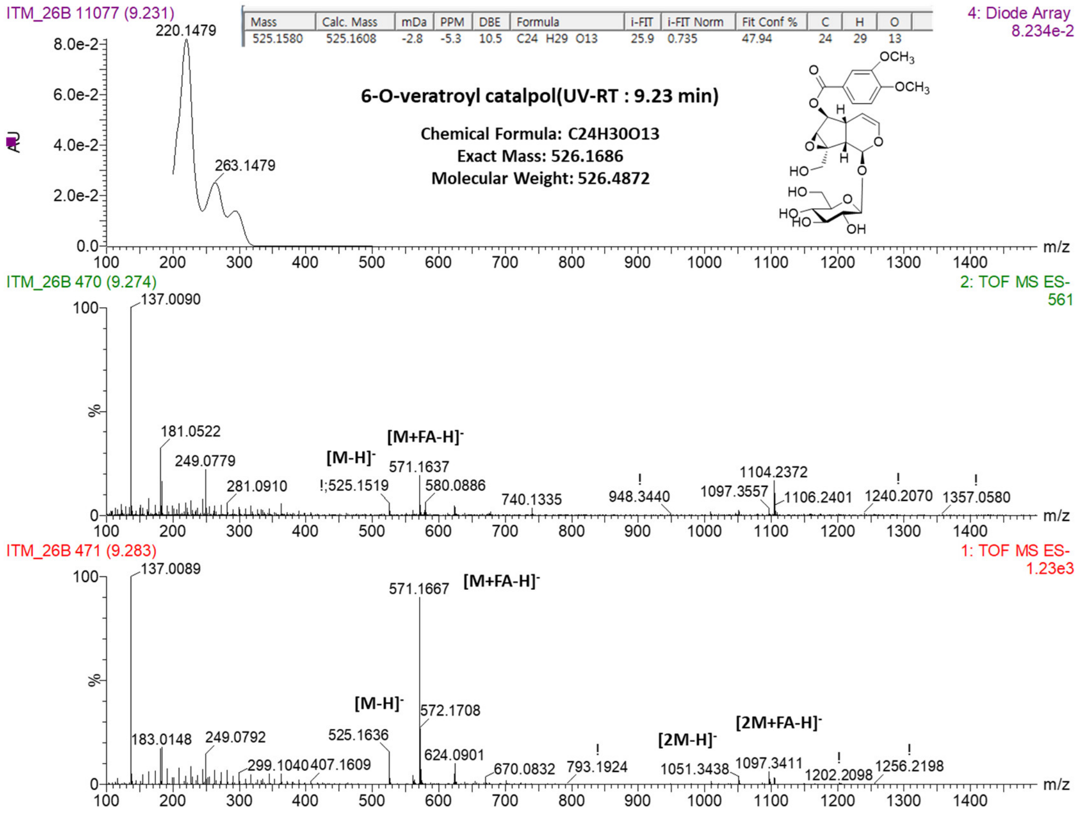

Figure S2-6. UV, MS/MS, MS and HREIMS data of 6-*O*-Veratroyl catalpol (**5**).

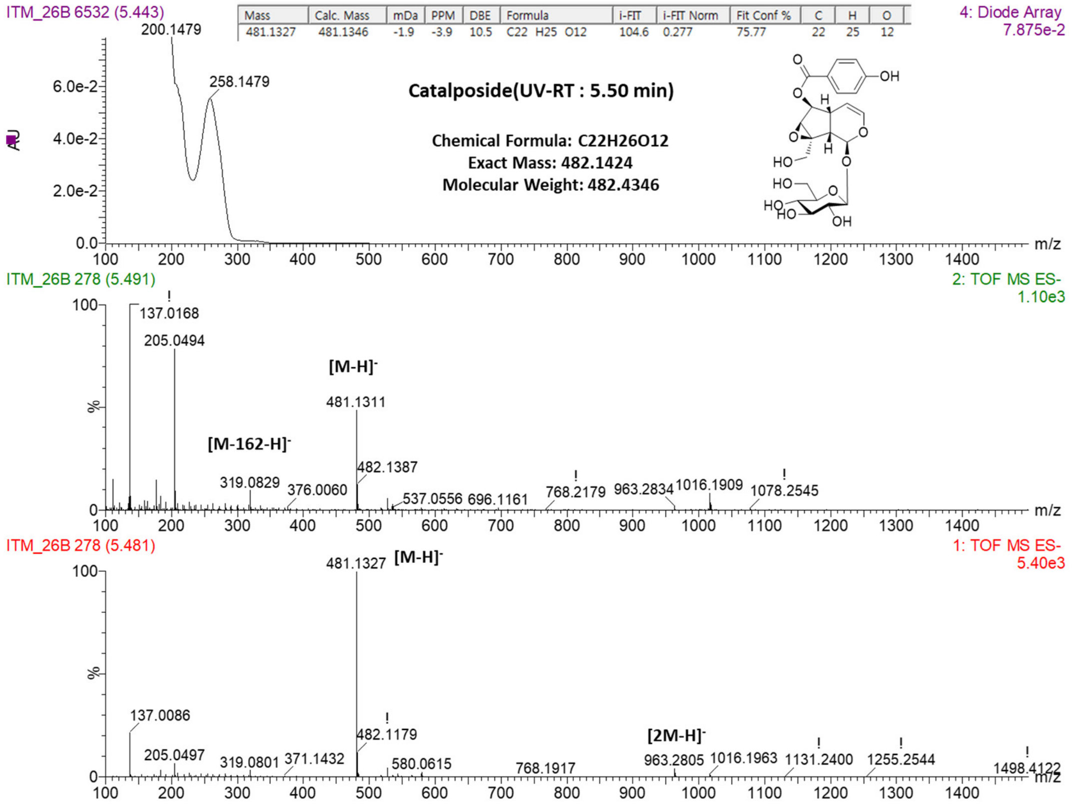

Figure S2-7. UV, MS/MS, MS and HREIMS data of Catalposide (**6**).

Western blot analysis

Western blots were performed as described in the "Materials and Methods" section. Here, we show the uncropped and unprocessed original image of the western blot image shown in our data figures.

A

Target	Size	
p-PKCδ	78 kDa	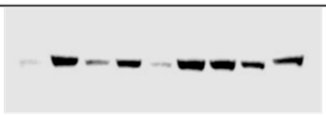
Tubulin	52 kDa	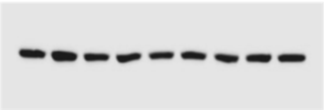

B

Target	Size	
EGR-1	82 kDa	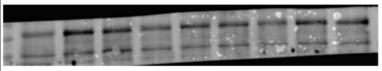
Tubulin	52 kDa	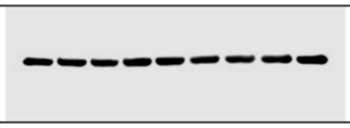

Figure S3. Original western blots in Figure 5. (A) Uncropped and unprocessed images of Figure 5E. (B) Original images of Figure 5F.

A

Target	Size	
p-PKC (pan)	78 kDa	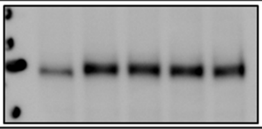
p-PKCδ (Thr505)	78 kDa	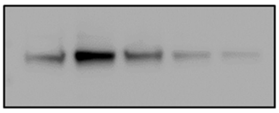
p-PKCθ (Thr538)	78 kDa	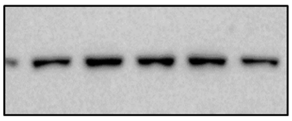
p-PKCα/βII (Thr638/641)	78 kDa	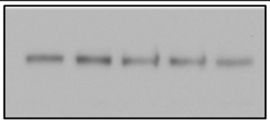
p-PKCμ (Ser744/748)	129 kDa	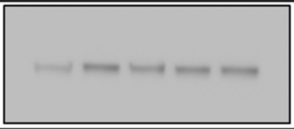
Tubulin	52 kDa	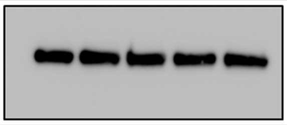

B

Target	Size	
p-PKCδ	78 kDa	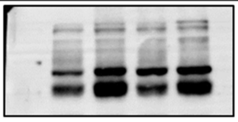
PKCδ	78 kDa	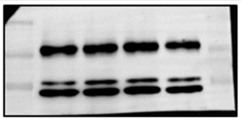
p-ERK	40/42 kDa	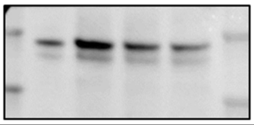
ERK	40/42 kDa	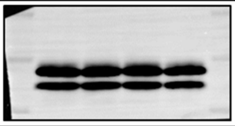
EGR-1	82 kDa	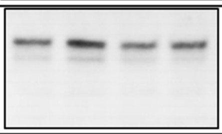
Actin	52 kDa	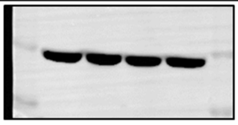

Figure S4. Original western blots in Figure 6. (A) Uncropped and unprocessed images of Figure 6A. (B) Original images of Figure 6B.

Methods for Supplementary Table S1

Protein structure and compound preparation for computational calculations

We prepared protein kinase of PKCδ for molecular docking simulation. Since the PKCδ structure is unavailable in Protein Data Bank (PDB), we performed a homology modeling simulation based on the given PKCδ sequence via PQR-SA (Pseudo Quadratic Restraint with Simulated Annealing)[PMID: 22648914]. The best template structure of 3PFQ was used with a total of 10 template structures to generate homology restraint potentials (distances and dihedral angles), and the potent maximum sequence identity from template structures is 0.84. We performed a simulated annealing method as a global optimizer with the generated restraint potentials to get the energy optimal structure. We picked the best energy structure from the generated structures. The CHARMM program (Chemistry at HARvard Macromolecular Mechanics: http://www.charmm.org) was used for these processes. For docking simulations using the structure of the six compounds, the 2D structure of each compound was downloaded from the PubChem site (https://pubchem.ncbi.nlm.nih.gov), and the 3D structure for each compound was formed using the MarvinSketch program (ChemAxon; http://www.chemaxon.com; 5.11.4, 2012).

Protein-compound docking simulations

Protein-compound docking simulations were conducted using the AutoDock Vina program (http://vina.scripps.edu). In order to select the docking position of the compounds, the Cα coordinate of the protein backbone was used as the docking center. The box size of the docking grid was 20Å×20Å×20Å. Flexible ligand docking simulations were performed 20 times with different random seeds in each box. After the compounds were docked at each docking center, ligand clustering was performed again to classify the docked ligand structures by orientations and positions. The ligand clustering calculated the ligand's center of geometry (COG) and classified the ligand where the COG is located within 1Å. In order to select the ligand structure with the lowest binding energy in each cluster, the free energy (ΔG = lowest energy+($-kT\ln N$), where k is Boltzmann constant, T is room temperature

(300K), and N is the number of cluster structures) reflecting the population size was calculated. The lowest energy means the lowest docking energy in each cluster. After that, we sorted them in the order of the docking energy, and the results, including the best two structure energies, are tabulated in **Table S1**.

Table S1. Molecular docking energy of PKCδ with six components (**1-6**)

	**Compounds**	**Docking energy in kcal/mol between PKCδ and compounds**	
		The lowest energy	The second-lowest energy
**1**	Piscroside C	-7.2[a] (ATP)[b]	-7.1 (ATP)
**2**	Verproside	*-7.5 (Surface)*	*-7.4 (ATP)*
**3**	Isovanillyl catalpol	-7.3 (Near)	-7.2 (ATP)
**4**	Picroside II	-7.3 (ATP)	-7.2 (ATP)
**5**	6-*O*-Veratroyl catalpol	-7.3 (ATP)	-7.0 (ATP)
**6**	Catalposide	-7.4 (ATP)	-7.0 (ATP)

[a] two energies are tabulated (the lowest and the second-lowest energy). [b] the locations of the compound on the kinases. ATP: a chemical on the ATP binding site. Near: a chemical near the ATP binding site (it could be inhibitory on the entrance). Surface: a chemical on a surface far from the ATP binding site (it could be allosteric inhibition). A molecular docking simulation shows that verproside has the lowest docking energy (italics), suggesting that verproside directly binds to PKCδ.

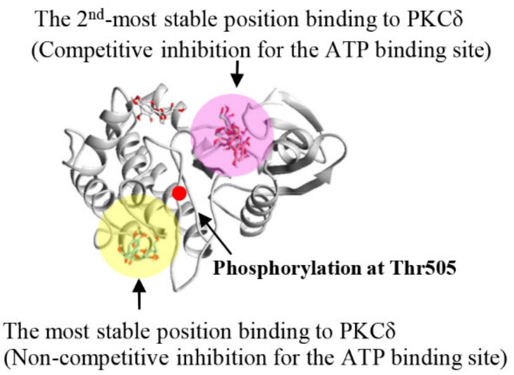

Figure S5. Molecular docking simulation of PKCδ and verproside. (A) The PKCδ homology model structure is depicted as a gray cartoon image. The adenosine triphosphate (ATP)-binding site is shaded pink. Verproside is drawn as a stick model colored in red or green. Phosphorylation of PKCδ at Thr505 is marked with a red spot. Two positions in PKCδ were predicted as direct binding sites for verproside:1) the ATP-binding site (shaded in pink) and 2) the surface area far from the ATP-binding site (shaded in yellow). The most stable binding between PKCδ and verproside was observed on the yellow surface of PKCδ (Table S1, red

letters), suggesting its effect on PKCδ activity could involve allosteric inhibition at the ATP-binding site (pink area) or at phosphorylation sites such as Thr505.
